# Multispectral Deep Neural Network Fusion Method for Low-Light Object Detection

**DOI:** 10.3390/jimaging10010012

**Published:** 2023-12-31

**Authors:** Keval Thaker, Sumanth Chennupati, Nathir Rawashdeh, Samir A. Rawashdeh

**Affiliations:** 1 Electrical and Computer Engineering, University of Michigan-Dearborn, Dearborn, MI 48128, USA; sumchenn@amazon.com (S.C.); srawa@umich.edu (S.A.R.); 2 Department of Applied Computing, Michigan Technological University, Houghton, MI 49931, USA; narawash@mtu.edu

**Keywords:** multispectral fusion, RGB-T fusion, low-light object detection

## Abstract

Despite significant strides in achieving vehicle autonomy, robust perception under low-light conditions still remains a persistent challenge. In this study, we investigate the potential of multispectral imaging, thereby leveraging deep learning models to enhance object detection performance in the context of nighttime driving. Features encoded from the red, green, and blue (RGB) visual spectrum and thermal infrared images are combined to implement a multispectral object detection model. This has proven to be more effective compared to using visual channels only, as thermal images provide complementary information when discriminating objects in low-illumination conditions. Additionally, there is a lack of studies on effectively fusing these two modalities for optimal object detection performance. In this work, we present a framework based on the Faster R-CNN architecture with a feature pyramid network. Moreover, we design various fusion approaches using concatenation and addition operators at varying stages of the network to analyze their impact on object detection performance. Our experimental results on the KAIST and FLIR datasets show that our framework outperforms the baseline experiments of the unimodal input source and the existing multispectral object detectors.

## 1. Introduction

While great strides have been made in computer vision in recent years with the advent of deep learning, object detection in inclement weather and low-illumination conditions remains a challenging perception task for autonomous driving [1,2,3]. Although overall traffic-related fatalities have declined in the US over the last few decades, pedestrian fatalities have steadily increased. In 2019, 3 out of 4 pedestrian fatalities occurred after dark [4]. Most of the current object detection algorithms are targeted to the benchmarks for color images with good illumination, whereas where they tend to decline in performance is under low illumination and inclement weather conditions.

All objects emit thermal energy, also known as a heat signature. Thermal cameras detect heat signatures to compose an image. Consequently, thermal cameras are inherently immune to spectral illumination variability. While RGB cameras provide high texture details with spatial resolution, infrared cameras distinguish active targets from their background based on the radiation signals. The fusion of RGB and IR images have shown improvement in pedestrian detection [5,6]. In addition, thermal cameras have recently become popular for autonomous driving and surveillance applications due to a decline in sensor prices. Thus, robust detection and classification of objects in the multimodal domain is an important problem to be addressed for deployment in the real-world environment.

Image fusion is an image enhancement technique that combines images from different modalities to generate an informative image. The image fusion process can be classified into three different processes: pixel-, feature-, and decision-level fusion. Pixel-level fusion combines the original information in the source images [7]. Choi et al. [8] performed pixel-level image fusion using a joint bilinear filter to fuse RGB and IR images. In feature-level image fusion, features such as edges and textures are identified for fusion [9]. Decision-level fusion combines results from multiple algorithms to yield a final decision. Torresan et al. [10] detected pedestrians in thermal and visible images independently, and the information was fused at the decision level through a final merging and validation process.

**Figure 1:**
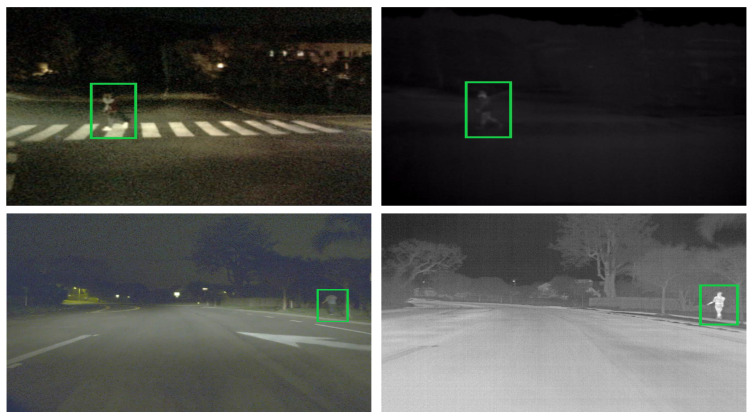
(**Left** column): The visual images from the KAIST and FLIR datasets provide distinctive visual features; however, in low lighting, human silhouettes are more apparent in the infrared domain (**Right** column).

Object detection has witnessed significant breakthroughs in recent years due to the introduction of frameworks such as Faster R-CNN [11] and YOLO [12]. These models rely on large-scale datasets such as MS-COCO and ImageNet for training. The combination of large datasets and frameworks have demonstrated significant performance improvement in the RGB domain; however, similar success in the thermal domain has been restricted due to lack of availability of large-scale thermal datasets. Intuitively, we can observe from Figure 1 that the fusion of infrared and RGB images would provide complementary information in challenging weather conditions, especially since thermal imaging is more robust against illumination variability, as well as weather conditions involving rain, fog, or snow.

Inspired by the recent success of deep learning (DL)-based object detectors, we exploit existing DL-based models to extend similar success for multimodal object detection. In this paper, we present a fusion framework based on Faster R-CNN and feature pyramid networks (FPNs) [13]. Our proposed framework fuses visual and infrared feature maps using a concatenation operation. Our ablation experiments on the concatenation and addition operator are motivated by the intention to understand the performance impact of fusion operators that would be applicable to similar multimodal fusion applications. We also implemented a squeeze and excitation layer [14], which has shown performance improvement by adaptively adjusting the weighting of the feature maps. We perform a comprehensive set of experiments on the KAIST and FLIR datasets and evaluate them using popular object detection metrics, including mean average precision (mAP) and the log-average miss rate.

The remainder of the paper is organized as follows: Section 2 provides a brief overview of related multimodal image fusion approaches. Section 3 describes our model architecture and parameters. Section 4 discusses the dataset, experimental setup, and discussion. Lastly, this paper concludes in Section 5.

## 2. Related Work

Driven by the success of convolutional neural networks (CNNs) in the last few years, multimodal image fusion has gained significant traction in the research community. Object detection in the thermal domain has been an active area of research for military and surveillance applications even before deep learning gained popularity. One of the early works on person detection using visual and infrared imagery was presented by Krotosky et al. [15]. The framework computed a probabilistic score for evaluating pedestrian presence using the histogram of oriented gradients (HOG) and a support vector machine (SVM). The detector utilized color and infrared features individually, and outputs were combined for a unified detection framework. Davis et al. [16] employed a two-stage template-based algorithm for person detection. A fast-screening procedure with a generalized template identified a potential area of interest, and AdaBoosted ensemble classifiers were used to test the hypothesized person locations. Teutsch et al. [17] proposed a two-stage person detection model using hotspots classification. The implementation of maximally stable external regions (MSERs) identified the hotspots. These hotspots were verified using the discrete cosine transform (DCT) and a modified random naïve Bayes (RNB) classifier.

The introduction of the KAIST multispectral dataset by Hwang et al. [6] revived CNN-based multispectral pedestrian detection. The proposed pedestrian detection is an extension of aggregated channel features (ACFs). The ACFs detector operates in a sliding window, and it generates channel features from subsampled and filtered channels. The extension of ACFs incorporates a contrast-enhanced version of the thermal images and uses the HOG to generate combined feature maps. The classification of the person class is conducted using boosted decision trees (BDTs). An early application of CNN-based multispectral person detection was presented by Wagner et al. [18]. They investigated both early- and late-fusion using CNN-based methods, with late-fusion methods demonstrating superior performance compared to the ACF+T+THOG-based solutions of that time. Choi et al. [19] generated region proposals separately on visual and infrared images first and applied support vector regression (SVR) on top of concatenated convolutional features to obtain classification.

Li et al. [20] proposed illumination-aware Faster R-CNN (IAF R-CNN) that integrates color and thermal subnetworks through a weighting mechanism to boost the final detection performance under varying illumination conditions. Xu et al. [21] employed crossmodality learning through a nonlinear mapping to model the relation between visual and infrared images. On the second stage, the feature representations are transferred to a secondary deep network, which uses visual images as an input for detections. The other notable study includes Devaguptapu et al. [22], who proposed a pseudo-multimodal object detector that uses a well-known image-to-image translation framework to generate pseudo-RGB images from thermal images. The multimodal Faster R-CNN architecture used a concatenation operator to fuse pseudo-RGB and thermal images.

Additionally, Yadav et al. [23] developed a two-stream VGG-16 encoder to extract visual and thermal features, thereby merging the resultant feature maps at a mid level. In the broader context of multispectral fusion methodologies, which typically encompass early, late, or learnable fusion, an insightful study on the performance implications of varying fusion positions was conducted by Liu et al. [24]. The investigation involved early, mid, and late fusion on the Faster R-CNN network with a VGG-16 backbone. Feature maps were fused using the concatenation operator, and a network in network (NIN) was implemented through a 1 × 1 convolution layer. The findings revealed that mid-level fusion consistently achieved superior performance compared to early or late fusion approaches. While recent years have witnessed the introduction of various CNN-based architectures, such as feature pyramid, thereby addressing the challenge of object handling at different scales, and squeeze and excitation networks, thereby demonstrating noteworthy accuracy gains through channelwise attention, the optimal fusion positions for ensuring similar accuracy enhancements remain less clear. Given the limited exploration of these optimal fusion positions, our study delves into investigating the impact of varying fusion positions, operators, and their overall influence on the fusion process.

## 3. Proposed Method

There have been several multispectral object detectors introduced in the last few years, some of which have been discussed in Section 2. In this section, we introduce our deep learning-based multispectral object detector in detail. Our model is based on the Faster R-CNN framework with the addition of FPNs. While low-cost object detectors such as YOLO or SSD networks have demonstrated comparable accuracy against region-based detectors such as Faster R-CNN, they exhibit difficulty in detecting smaller objects. Furthermore, FPN has demonstrated enhanced accuracy regarding objects at different scales. For instance, incorporating FPN into RPN led to an 8-point improvement in average recall compared to the RPN baseline, and there was a notable 12.9-point boost in performance in detecting small objects in the MS-COCO dataset. The FPN builds high-level semantic feature maps at all scales by combining feature maps from different levels of the feature extractor.

**Figure 2:**
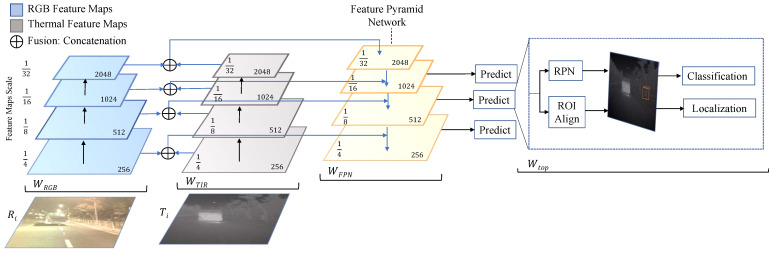
Architecture of the proposed multispetral fusion network. The shared ResNet-50 backbone extracts feature maps from visual and infrared images. The feature maps are fused using a concatenation operator prior to being processed in the feature pyramid network, and prediction is obtained from classification and regression layers.

The Faster R-CNN model consists of two main modules: the region proposal network (RPN) and the Faster R-CNN network for object detection and classification. RPN is a fully convolutional network that proposes background and foreground objects and their corresponding objectness score. Since the RPN provides region proposals of difference sizes, Faster R-CNN uses a region of interest (ROI) pooling layer, which normalizes different proposals to a fixed size before being processed through the classification and regression layers. The overall proposed methodology for multispectral object detection is summarized in Figure 2 and also complimented by the Algorithm 1 below.
**Algorithm 1:** Proposed Methodology  
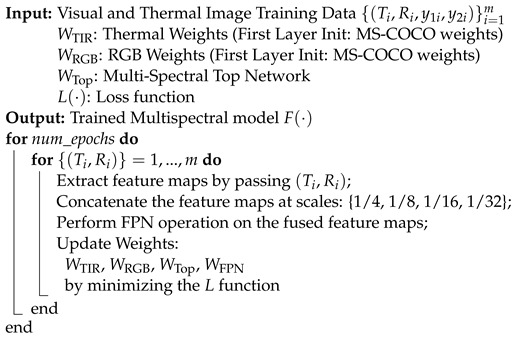


**Figure 3:**
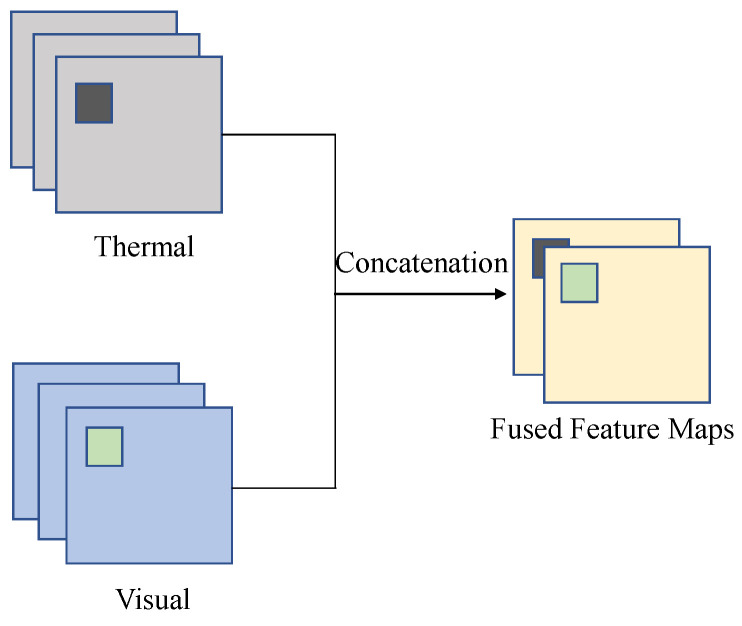
Sensor modality fusion using concatenation operator.

The key idea of our methodology is to use a shared ResNet backbone between the thermal and visual channels and to fuse the channel features using a concatenation operator prior to the pyramid networks. We modify the Faster R-CNN network to incorporate both modalities and integrate the feature pyramid network within the feature extraction backbone. As depicted in Figure 2, a common ResNet-50 backbone extracts multiscale feature maps. The shared ResNet-50 encoder outputs multiple scales of feature maps {1/4, 1/8, 1/16, and 1/32} with respect to the original input images. As illustrated in Figure 3, the concatenation operator is utilized to combine feature maps from both modalities.

The fused feature maps from the concatenation operation in the scales of 1/4 to 1/32 are consumed in a top-down fashion and output 256 channels while maintaining the original input scale. Finally, the feature maps from different levels are added and passed on to the prediction heads. The configuration parameters for the RPN, ROI pooling, classification, and localization layers remain consistent with the default implementation [11]. The classification and regression loss function within the RPN is defined as follows:

(1) $$L\left(\left\{p_{i}\right\},\left\{t_{i}\right\}\right)=\frac{1}{N_{\mathrm{cls}}}\sum_{i}L_{\mathrm{cls}}\left(p_{i},p_{i}^{*}\right)+\lambda \frac{1}{N_{\mathrm{reg}}}\sum_{i}p_{i}^{*}L_{\mathrm{reg}}\left(t_{i},t_{i}^{*}\right)$$

where *i* is anchor index in minibatch, and the classification log loss is computed for predicted probability, $p_{i}$, of an anchor being an object over $p_{i}^{*}$ ground truth. The regression loss is only computed for positive anchors, which uses a smooth L1 norm function. The $t_{i}$ represents coordinates of the predicted bounding box, and $t_{i}^{*}$ is the ground truth bounding box associated with a positive vector. The λ parameter is used such that both therms are roughly equally balanced.

We now discuss the classification loss function, which uses the crossentropy loss. The crossentropy loss measures the performance of a classification model whose output has a probability value between 0 and 1. Crossentropy loss increases as the predicted probability diverges from the actual reference value. As presented in Equation (2), crossentropy loss is measured over k classes for all pixels in the image, where $\hat{y_{i}}$ is the prediction probability, and $y_{i}$ is the ground truth.

(2) $$Loss=-\sum_{i=1}^{k}y_{i}\times log\left(\hat{y_{i}}\right)$$

## 4. Experiments, Results, and Discussions

### 4.1. Experimental Setup

Our experiments were conducted on the KAIST and FLIR datasets. The KAIST multispectral dataset, released in 2015, provides over 95.3 k pairs of visual and infrared images. The dataset consists of over 50.2 k training images and 45.1 k testing images with 41.5 k and 44.7 k pedestrian labels, respectively. The well-aligned image sets are captured at 640 × 480 resolution using a FLIR A35 camera with a day and night split. We sampled every 2nd frame from the training set, as outlined by König et al. [25]. The testing set samples every 20th frame, which contains 2252 images with approximately 797 night scene images. In addition, we evaluated the results by sampling every single frame from the testing set.

The experiments were also evaluated on the FLIR dataset released by FLIR systems. The dataset comprises 60% daylight and 40% night scene images captured at 640 × 512 resolution using a FLIR Tau2 camera. Although the dataset provides synchronized visual and infrared images, the alignment between the paired images differs. The dataset includes over 8.8 k training and 1.2 k testing images. For the experiments, we evaluated the results on person, car, and bicycle classes with total annotations of 28 k, 46 k, and 4.4 k, respectively. Due to the unavailability of separate day and night split test sets in the FLIR dataset, our experiments were evaluated on the provided validation set.

The experiments were conducted using the MMDetection toolkit based on the PyTorch framework. We trained our model on full-resolution images for both datasets and used batch normalization with a batch size of 16 images. We used stochastic gradient descent (SGD) as an optimizer with a learning rate of 0.001, momentum of 0.9, and weight decay of 10^−4^. The ResNet encoders in our model were initialized with weights from the MS-COCO dataset and trained on the networks for 16 epochs in all experiments. The experiments were trained using Google Colaboratory with a Tesla P100 GPU (16 GB RAM).

The performance outcomes of our model and experiments were evaluated using the widely popular object detection metrics: mean average precision (mAP) and log-average miss rate (MR). We used an intersection over union (IoU) threshold of 0.5. Hence, a detected bounding box with a threshold over 50% will be considered as a true positive if it successfully matches the ground truth, whereas an unmatched detected bounding box and unmatched ground truth detection are considered false positives and false negatives, respectively. We utilized the log-average miss rate metric to compare different detectors. The log-average miss rate is computed by averaging the miss rate (false negatives) at a nine false-positive-per-image (FPPI) rate evenly spaced in the log-space in the range of 10^−2^ to 10^0^.

### 4.2. Results

#### 4.2.1. Baseline

Table 1 below demonstrates the training results on the KAIST dataset with evaluation on every single frame, as well as every 20th frame. The experiments were trained on both imagery independently using the Faster R-CNN and with integration of the FPN as an addition. The hyperparameters for all experiments are as defined in Section 4.1. In addition, we used the results from MMTOD [22] for our baseline comparison. The best-performing model from MMTOD was initialized with MS-COCO weights for both datasets. We observed that thermal imagery trained on the Faster R-CNN with FPN yielded the highest mAP score.

#### 4.2.2. Proposed Method

As outlined in the earlier section, our proposed method uses RGB and thermal imagery as inputs into our model. The shared backbone between both imagery fuses the feature maps using a concatenation operation prior to being processed in the feature pyramid network. As seen in Table 1, we observe that our method outperformed the baseline RGB-T networks, as well as the baseline network of a single input source. Similarly, we observe that our proposed method outperformed the baseline RGB-T detector on the KAIST and FLIR datasets.

#### 4.2.3. Ablation Studies

Due to a lack of studies involving varying fusion positions with concatenation and addition operators, we devised a thorough set of experiments to analyze the performance impact with respect to varying fusion approaches to study the effectiveness of the merging operators. The experiments fused the feature maps from both modalities using concatenation, addition, and a 1 × 1 convolution filter. Additionally, we implemented a squeeze and excitation layer, which has been demonstrated to be an effective approach to adaptively adjust the weighting of the feature maps. The fusion positions, ‘Pre’ and ‘Post’ in our experiments indicate application of the merging operator prior to being processed through the feature pyramid networks. For instance, the fusion method of concatenation with a 1 × 1 filter, a fusion position of Post-FPN, and an SE position at post would indicate that both modalities are merged after FPN operation, and a subsequent SE layer is implemented. From Table 2, we observe that fusion at post-FPN with a concatenation operator and a 1 × 1 convolution filter achieved the highest mAP score among all experiments while retaining the less learnable parameters compared to other concatenation methods. In the FLIR dataset, we observe a marginal performance impact with respect to the mAP score.

We used an addition operator, which is an alternative merging operator for fusing feature maps. Similar to concatenation experiments, the addition experiments involved feature maps fusion at the pre- and post-FPN process. The squeeze and excitation layer was also implemented to further analyze the performance impact on object detection in the multimodal domain. The addition experiments in Table 3 demonstrated comparable mAP scores to the concatenation operators while requiring less learnable parameters than the concatenation operator.

### 4.3. Discussion

#### 4.3.1. Qualitative Results Comparison

**Figure 4:**
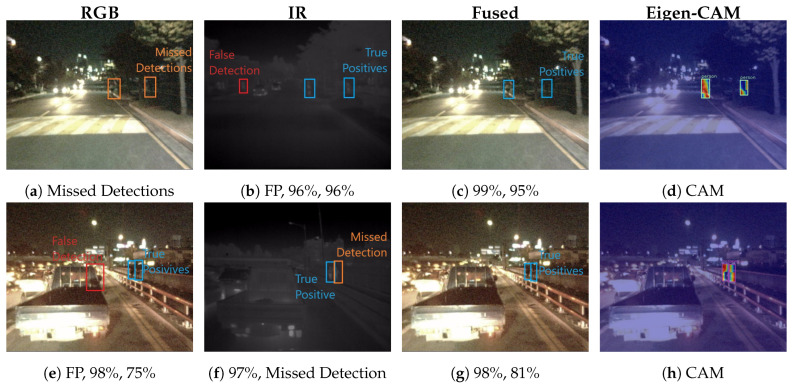
Qualitative comparison of results: Enhanced detection performance is evident in the multispectral domain, where instances of both missed detections and false positives can be observed in the visual and infrared spectra.

In Figure 4, we demonstrate sample night scene images from the KAIST dataset. In the top row images, we observe a notable discrepancy in the detection performance between the RGB and infrared domains. Specifically, detections were missed in the RGB domain, whereas the person instance was correctly identified in the infrared domain; however, a false positive was also detected. Fused features from the visual and infrared domain demonstrate detection with higher confidence compared to infrared. Additionally, we visualized the class activation map using Eigen-CAM [26] in the multimodal domain, which confirms the localized objects with respect to weights.

As seen in the bottom row of images, the RGB domain captured two detections accurately, but it also registered a false positive. In contrast, the infrared domain successfully discarded the false positive but missed a true detection. This trade-off between domains becomes evident, thereby showcasing the improved accuracy in the multispectral domain as a result of complementary information. Appendix A provides supplementary qualitative comparisons, thus encapsulating imagery samples with day and night scenarios.

#### 4.3.2. Detection Benchmark under Image Corruption

Object detection in real-world scenarios requires robust performance under diverse weather conditions. To evaluate the robustness of our proposed model, we investigated its performance under varying weather conditions using image corruption methods developed by Hendrycks and Dietterich [27]. Their work demonstrated that convolutional neural networks (CNNs) often fail to generalize beyond the training data distribution. Michalis et al. [28] demonstrated that robustness benchmarking drops by 30–60% of the original performance when subjected to varying noises and corruptions.

To assess the robustness of our model under different weather conditions, we employed three types of image corruptions: fog, frost, and snow. We evaluated the model’s performance with respect to the RGB, IR, and RGB-T (proposed) models. We applied the most reasonable severity level (1), simulating real-world conditions, and measured the average precision at 50% IoU. We trained each model on the respective corruption type and evaluated its performance under both day and night conditions as shown in the Table 4. As expected, we observed a significant impact on the average precision (AP) under varying weather conditions. However, we noticed that our RGB-T model retained, on average, higher average precision compared to the unimodal input sources. We attribute the RGB-T model’s performance gain to the infrared imagery’s ability to ignore textures and focus on object shapes. To further improve the model’s AP under varying distortions, we recommend employing data augmentation using stylized imagery, as described by Michalis et al.

#### 4.3.3. Is Multispectral Fusion Really Complementary?

We analyzed the complementary potential of object detection from visual and infrared fusion through various experiments conducted on both imagery types. First, visual and infrared images were trained independently using the Faster R-CNN network with the addition of a feature pyramid network as a baseline. To study the effectiveness of the multispectral fusion, we compared the baseline results against our multispectral neural network that uses visual and infrared images as input. The training parameters were kept constant between all our experiments. The visual and infrared images were trained on the KAIST dataset using the provided images sets for day and night scene images.

For testing, we sampled every single frame from its respective day and night scene image sets. As shown in the Table 5 during daytime, we observe that thevisual images outperformed infrared images, as would be expected due to the high spatial resolution in the visual images. In contrast, we observe improved detection in thermal images at night due to thermal images providing better visual features. However, we observe our multispectral network to have outperformed with respect to both day and night scene images based on the mAP and MR metric. The miss rate of 28.7% was significantly lower compared to its visual and infrared counterparts.

#### 4.3.4. State-of-the-Art RGB-T Detectors Comparison

**Figure 5:**
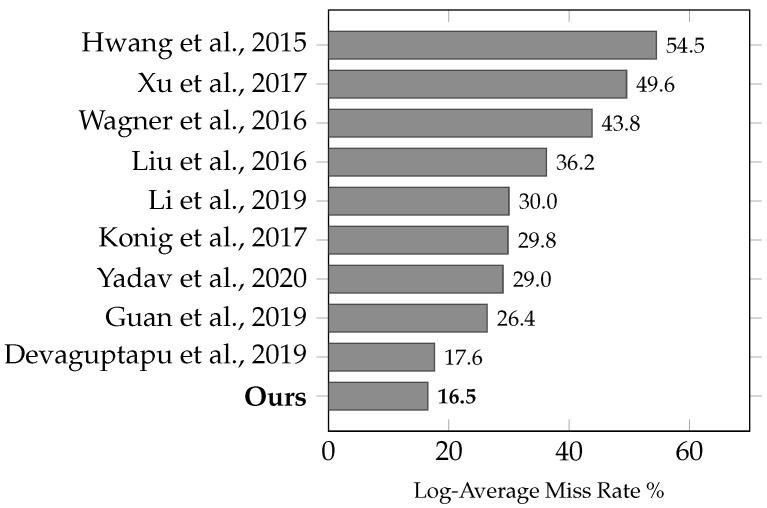
State-of-the-art RGB-T Detectors [6,18,20,21,22,23,24,25,29].

We compared the MR with the other published reports under reasonable configurations [6], which provide a representative subset of the larger proposed dataset. The subset contains pedestrian annotations larger than 55 pixels. As shown in Figure 5, our results are compared with [29], as well as with the other architectures discussed in Section 2. The authors provided either codes or detections, on which we evaluated and reported their performance based on the improved annotations for the KAIST dataset. It can be observed that our model outperforms the current state-of-the-art RGB-T detectors and has achieved the lowest MR of 16.49%. In addition, our proposed method of a shared backbone between visual and infrared images is less computationally intensive compared with previous approaches.

## 5. Conclusions

In this study, we presented a multispectral object detection framework designed to improve detection capabilities in the multimodal domain. Our architectural approach, based on the Faster R-CNN algorithm and feature pyramid networks, seamlessly incorporates color and thermal channels into a unified network. We assessed the performance of our network using the KAIST and FLIR datasets. Through the experiments with the low-cost object detector, YOLO, we demonstrated that feature pyramid networks vastly improve accuracy. Additionally, we delved into an exploration of various fusion approaches to analyze the impact of fusion operators and fusion positions. Despite a minimal performance impact observed from ablation experiments, a comprehensive analysis of varied fusion positions and operators is prudent to ensure optimal object detection performance in the multimodal domain involving visual and infrared imagery. Our extensive empirical analysis demonstrates that our framework improves performance compared to the baseline and the current state-of-the-art RGB-T detectors.

## Figures and Tables

**Table 1:** Comparison of the baseline models with our method on KAIST and FLIR datasets. The KAIST dataset was trained on every 2nd frame using the improved annotations.

	Input	Model	mAP@0.5		
			KAIST		FLIR
			Eval-01	Eval-20	-
Baseline	RGB	YOLO	40.3	42.7	63.6
		Faster R-CNN	53.3	53.2	57.5
		Faster R-CNN w/FPN	53.2	53.1	71.9
	Thermal	YOLO	42.3	41.6	67.4
		Faster R-CNN	44.8	44.1	67.2
		Faster R-CNN w/FPN	48.2	48.0	79.3
	RGB-T	Faster R-CNN [22]	-	53.5	61.4
**Proposed**	RGB-T	Faster R-CNN w/FPN Fusion: Concat pre-FPN	**57.8**	**57.9**	**78.9**

**Table 2:** Ablation experiment—concatenation operator.

Fusion Method	Fusion Position	SE Position	mAP@0.5		Params (M)
			**KAIST**	**FLIR**	
Concat	Post-FPN	-	58.4	79.1	55.7
Concat-1 × 1	Pre-FPN	-	58.6	**79.5**	52.2
	Post-FPN	-	**60.4**	78.4	**41.7**
	Pre-FPN	Pre	58.0	79.4	53.6
	Pre-FPN	Post	58.3	79.2	52.9
	Post-FPN	Pre	58.7	79.2	41.8
	Post-FPN	Post	57.2	79.4	41.8

**Table 3:** Ablation experiment—addition operator.

Fusion Method	Fusion Position	SE Position	mAP@0.5		Params (M)
			**KAIST**	**FLIR**	
Addition	Pre-FPN	-	**59.0**	79.1	**41.1**
	Post-FPN	-	58.2	79.2	41.1
	Pre-FPN	Pre	**59.0**	**79.5**	42.5
	Pre-FPN	Post	56.6	**79.5**	41.8

**Table 4:** Weather corruption benchmark at IoU of 0.5 (AP at 50).

Model	Day				Night			
	Clean	Snow	Frost	Fog	Clean	Snow	Frost	Fog
RGB	56.9	17.0	15.9	18.9	41.0	11.2	11.8	14.7
IR	43.3	15.0	14.7	15.7	56.8	19.8	19.0	18.7
RGB-T (ours)	**58.9**	**17.6**	**18.4**	**19.0**	**60.4**	**20.5**	**19.5**	**20.2**

**Table 5:** Day–night mAP and log-average miss rate comparison.

Input	Day		Night	
	**mAP@0.5**	**MR**	**mAP@0.5**	**MR**
RGB	56.9	32.3	41.0	49.2
IR	43.3	46.8	56.8	46.8
RGB-T (ours)	**58.9**	**29.0**	**60.4**	**28.7**

**Figure A1:**
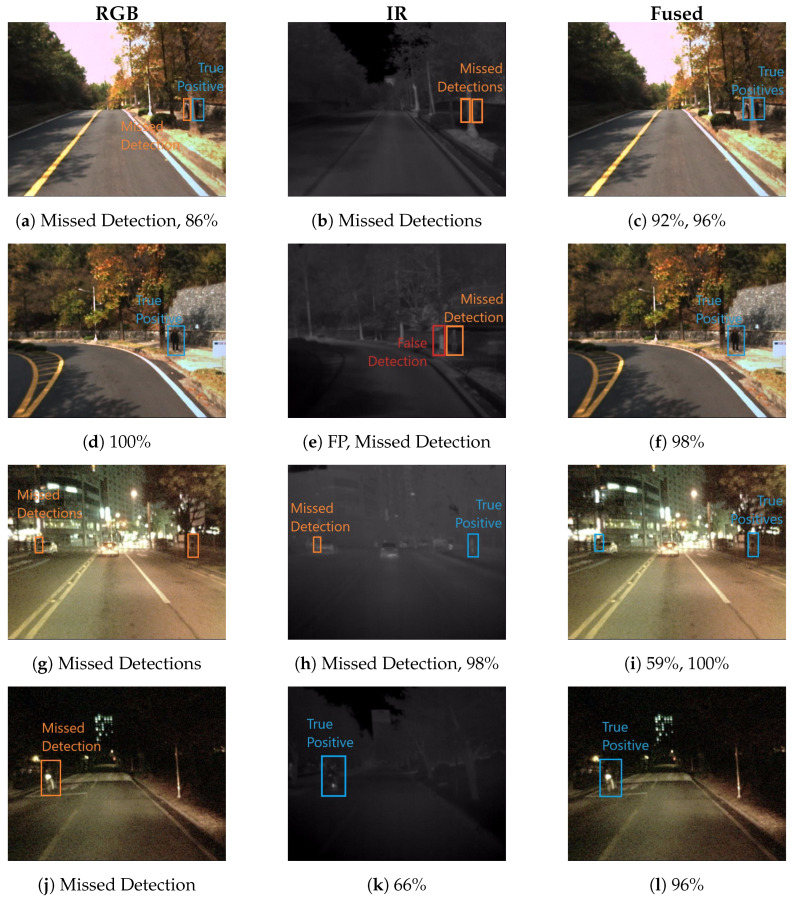
Additional qualitative comparison of results: Missed and false positive detections are represented by orange and red bounding boxes, respectively, while blue bounding boxes indicate accurate detections in accordance with the ground truth. Improved detection accuracy can be observed in the multimodal domain compared to missed detections in the visual domain.

## Data Availability

The study leverages publicly available datasets, specifically the KAIST and FLIR datasets, to enhance empirical analysis.
